# Separation Methods of Phenolic Compounds from Plant Extract as Antioxidant Agents Candidate

**DOI:** 10.3390/plants13070965

**Published:** 2024-03-27

**Authors:** Ike Susanti, Rimadani Pratiwi, Yudi Rosandi, Aliya Nur Hasanah

**Affiliations:** 1 Pharmaceutical Analysis and Medicinal Chemistry Department, Faculty of Pharmacy, Universitas Padjadjaran, Jl Raya Bandung Sumedang KM 21 r, Sumedang 45363, Indonesia; ike14001@mail.unpad.ac.id (I.S.); rimadani.pratiwi@unpad.ac.id (R.P.); 2 Faculty of Mathematics and Natural Sciences, Universitas Padjadjaran, Jl. Raya Bandung Sumedang KM 21, Sumedang 45363, Indonesia; 3 Drug Development Study Center, Faculty of Pharmacy, Universitas Padjadjaran, Jl. Raya Bandung Sumedang KM 21, Sumedang 45363, Indonesia

**Keywords:** isolation, separation, phenolic compounds, antioxidant, plant extract

## Abstract

In recent years, discovering new drug candidates has become a top priority in research. Natural products have proven to be a promising source for such discoveries as many researchers have successfully isolated bioactive compounds with various activities that show potential as drug candidates. Among these compounds, phenolic compounds have been frequently isolated due to their many biological activities, including their role as antioxidants, making them candidates for treating diseases related to oxidative stress. The isolation method is essential, and researchers have sought to find effective procedures that maximize the purity and yield of bioactive compounds. This review aims to provide information on the isolation or separation methods for phenolic compounds with antioxidant activities using column chromatography, medium-pressure liquid chromatography, high-performance liquid chromatography, counter-current chromatography, hydrophilic interaction chromatography, supercritical fluid chromatography, molecularly imprinted technologies, and high-performance thin layer chromatography. For isolation or purification, the molecularly imprinted technologies represent a more accessible and more efficient procedure because they can be applied directly to the extract to reduce the complicated isolation process. However, it still requires further development and refinement.

## 1. Introduction

Herbal medicine, also known as a phytopharmaceutical preparation is made exclusively from a whole plant or parts of plants. It can be manufactured in a crude form or as a purified pharmaceutical formulation [1]. Herbal products are readily available in the market. In Saudi Arabia, herbal medicine use was reported to range from 10.3% to 75.0% in 2019 [2]. In Indonesia, the most commonly used traditional medicine is for cancer or malignant tumors, with a prevalence of 14.4%. Joints/rheumatism and high cholesterol have the same prevalence of 11.3%. This is followed by stroke (10.2%), diabetes (9.9%), kidney disease (9.7%), and liver (8.0%). In China, pregnant individuals have been using Chinese herbal medicines for a long time. The usage rate is relatively high, with 65.7% of the population using them. Of this, 6.1% use them during pregnancy, while 55.6% use them after delivery [3].

In herbal medicine, secondary metabolites play a crucial role as they are responsible for the clinical effects [4]. Due to their diverse and specific biological activities, secondary metabolites are considered a valuable source of lead molecules for developing new drugs. Therefore, they are continuously being studied and explored for their potential in drug development. Some of these compounds even have the ability to act in additive or synergistic ways [5]. Phenolic compounds are secondary metabolites that have potent biological activity and are commonly found in various types of plants [6]. Phenolic compounds have many activities, such as antimicrobial [7,8,9], and anti-inflammatory effects and can aid in treating diseases like obesity [10], cancer [11], and diabetes [12], and are antioxidant [13,14,15,16,17]. 

**Figure 1:**
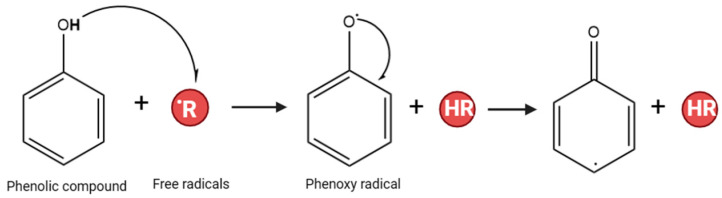
Mechanism action of phenolic compounds as an antioxidant agent.

Antioxidants play a crucial role in preventing the process of oxidation. Oxidation is a chemical reaction that can produce free radicals and cause chain reactions, which can lead to significant damage to cells in organisms [18,19]. As antioxidants, phenolic compounds can act as radical scavengers. The hydroxyl group on the phenolic ring can transfer its hydrogen atom to a free radical, forming a delocalized and stabilized unpaired electron, phenoxy radical, across the phenolic ring [20]. The stabilization by the resonance effect of the aromatic nucleus prevents the continuation of the free radical chain reaction [21] (Figure 1).

Free radicals, Reactive oxygen species (ROS) and nitrogen species (RNS), such as superoxide, hydroxyl, and nitric oxide radicals can cause DNA damage and oxidize lipids and proteins in cells in the biological system [22,23,24]. Excessive levels of ROS can lead to oxidative stress, which can cause changes in various organ systems. Oxidative stress occurs when there is an imbalance between ROS or highly reactive compounds and antioxidants, leading to disrupted redox processes, and molecular damage because of insufficient antioxidant function [25]. Excessive levels of ROS can cause neurotoxicity [26], myocardial hypertrophy and fibrosis [27], hepatocyte dysfunction and apoptosis [28], and insulin resistance [29]. An abnormal level of ROS was identified as a predisposing factor for cell transformation, triggering pro-oncogenic signaling pathways, altering gene expression, and causing genomic instability and DNA damage [25]. The DNA damage or mutation can lead to cancer [30]. The ROS/RNS induce inflammatory cells to damaged tissue sites, which can contribute to the progression of cancer (Supporting Information Figure S1). ROS/RNS cause oxidative stress and nitrosative stress, which are also activated by chronic inflammation that induces inflammatory cells and activates gene expression through various pathways [31]. Inflammatory cells discharge cytokines that initiate the oxidation and nitration of lipids, proteins, and carbohydrates. Furthermore, the transcription factors and cytokines elicit apoptosis, causing an imbalance between pro-apoptotic and apoptotic gene surroundings [31]. This imbalance results in regeneration and cell death, eventually leading to cancer through gene modifications, mutations, proliferation, angiogenesis, and other associated mechanisms [31].

Natural antioxidants, such as polyphenols or phenolic compounds, are widely distributed in food and medicinal plants and are known for their anti-inflammatory, anti-aging, anti-atherosclerosis, anticancer and antioxidant properties [32]. Hibiscetin-3- glucoside is a flavonoid compound isolated from the petals of *Hibiscus rosa* sinensis. Hibiscetin-3- glucoside has excellent great antioxidant activities, as compared to the standard ascorbic acid. The Hibiscetin-3- glucoside could be utilized for scavenging free radicals, preventing the formation of toxic products, and maintaining the shelf life of food and pharmaceuticals [33]. The ethyl acetate fraction of *Anacardium occidentale* L. (Anacardiaceae) leaf contained antioxidant compounds, such as agathisflavone, and a mixture of quercetin 3-O-rutinoside and quercetin 3-O-rhamnoside. The mixture of quercetin 3-O-rutinoside and quercetin 3-O-rhamnoside (2:1) was the most effective in scavenging free radicals in the DPPH assay, with an IC_50_ value of 0.96 ± 0.01 µg/mL. That mixture also exhibited the highest activities in the total antioxidant capacity (TAC) and ferric-reducing antioxidant power (FRAP) assay [34]. 

The process of developing bioactive compounds from natural products into drugs has remained challenging, starting from the screening of natural products, the isolation of bioactive compounds, the characterization and optimization of the bioactive compound, the determination of the mechanism of action, and pharmaceutical development [35,36]. The isolation of secondary metabolites is a critical step before biological characterization. It is also an essential method for obtaining compounds that are hard to synthesize or for which there are no commercial standards. Many isolation methods are used to isolate phenolic compounds that have antioxidant activity, including column chromatography [37], high-performance liquid chromatography (HPLC) [38], medium-pressure liquid chromatography (MPLC) [39], centrifugal partition chromatography (CPC) [40], high-speed counter-current chromatography (HSCCC) [41], and high-performance counter-current chromatography (HPCCC) [42]. Molecularly imprinted polymer techniques (MITs) have also been developed to selectively isolate phenolic compounds from extracts producing synthetic polymeric materials with homologous adsorption sites to a template molecule [43]. 

Phenolic compounds are known for their antioxidant properties and are widely distributed in natural products. Many reviews from 2021 to 2023 have highlighted the extraction of phenolic compounds in natural products [44,45] or phenolic compounds with antioxidant activity [46]. However, there are still only a few reviews that focus on the isolation methods of antioxidant phenolic compounds.

One such review was conducted by Shi et al. in 2022, in which they discussed the extraction, separation, and characterization methods, as well as the determination of antioxidant activity of phenolic compounds. The separation and characterization methods are membrane filtration, solid-phase extraction (SPE)-GC/LC (gas chromatography/liquid chromatography), liquid chromatography–mass spectrometry (LC-MS/MS), HPLC, capillary electrophoresis (CE), CCC, and CPC [21]. However, based on articles published in 2017–2023, several methods have not been discussed, including hydrophilic interaction chromatography (HILIC), supercritical fluid chromatography (SFC), MPLC, high-performance thin layer chromatography (HPTLC), and MITs methods. Therefore, this review aims to focus on the isolation methods for phenolic antioxidant compounds, including those not discussed in previous reviews.

This review will highlight the improvement in isolation methods for phenolic compounds with antioxidant activities using column chromatography, MPLC, HPLC, HILIC, CCC, SFC, MITs, and HPTLC as well as the combination of the methods to achieve more selective and efficient isolation methods. During the isolation process, a large amount of organic solvents like methanol, n-hexane, acetone, chloroform, benzene, and petroleum ether are required. However, the use of these solvents has various disadvantages, such as being flammable, explosive, poorly biodegradable, and toxic for the final product [47]. Thus, it is necessary to find an environmentally friendly and safe solvent to be used for the isolation process. This review also discusses using environmentally friendly green solvents for isolating, such as deep eutectic solvents (DES).

## 2. Phenolic Compound

**Figure 2:**
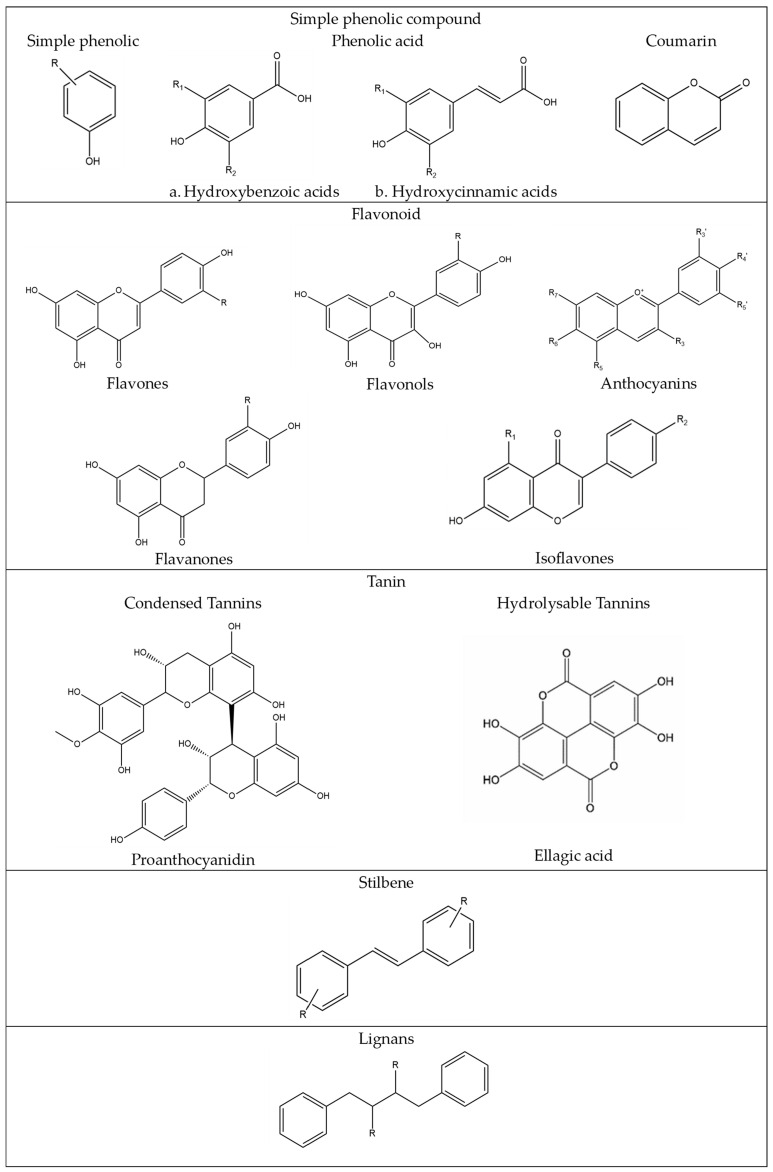
Classification of phenolic compounds: (1) simple phenolic compound, (2) flavonoid, (3) tannins, (4) stilbenes, and (5) lignans.

Phenolic compounds are natural metabolites that arise from the shikimate/phenylpropanoid pathway. This pathway directly provides phenylpropanoids, which are characterized by an aromatic ring with one or more hydroxyl substituents [48]. Figure 2 illustrates the various classes of phenolic compounds.

Simple phenolic compounds are substituted phenolic compounds with a C6 skeleton (Figure 2). The group, denoted by “R”, can be an organic group such as alkyl, alkenyl, aryl, hydroxy, alkoxy, amino, etc. It can be present in ortho (o), meta (m), or para (p) positions of the aromatic ring. There are three groups of simple phenolic compounds: simple phenolic, phenolic acids (hydroxybenzoic acids and hydroxycinnamic acids), and coumarins [49]. Simple phenolic compounds can be hydroxy phenols, dihydroxy benzenes, or trihydroxy benzenes. The compounds that belong to this classification are resorcinol, catechol, and pyrogallol [49]. Pyrogallol is a trihydroxy phenol isolated from the stem bark of Barringtonia asiatica. The pure compound exhibited significant biological activity, cytotoxicity, and antioxidant potential [50].

Phenolic acids are phenolic compounds that contain a carboxylic acid group [49]. Phenolic acids can be divided into two subgroups:

1. Hydroxybenzoic acids (Figure 2), the carboxylic acid group is directly attached to the phenol ring, the resulting phenolic compound (C6-C1). Examples: salicylic acid, protocatechuic acid, gallic acid.
2. Hydroxycinnamic acids (Figure 2), the carboxylic acid group and the phenol ring are separated by two doubly bonded carbon atoms (C6-C3). Examples: sinapic acid, ferulic acid, and caffeic acid [49,51].

Scopoletin or 7-hydroxy-6-methoxy coumarin is an example of a phenolic coumarin that contains two aromatic rings with a hydroxyl and methoxy group, as well as an oxo group [52]. Scopoletin can be isolated from many plants such as *Eupatorium laevigatum* [53], the root of *Hypochaeris radicata* [54], and *Lasianthus lucidus Blume* [54]. 

Flavonoids are a group of compounds that have (C6–C3–C6) as their basic skeleton, consisting of two aromatic rings connected to each other through a central three-carbon bridge [51]. Flavonoids are a diverse group of natural substances with phenolic structures that can be found in various sources including fruits, vegetables, grains, bark, roots, stems, flowers, tea, and wine [55]. The subclasses of flavonoids found in nature include anthocyanins, flavones, flavonols, flavanones, isoflavones, and flavanonols (Figure 2) [51]. For example, quercetin and kaempferol are the most common compounds in natural products. They are compounds that belong to the flavonols subclass. Quercetin and kaempferol are commonly found in polyphenols in fruits and vegetables. They are typically conjugated with sugar molecules in plants [56]. 

Tannins are present in various species across the plant kingdom, where they serve the purpose of safeguarding the plant against predators and potentially aiding in the regulation of plant growth. These tannins can be divided into two primary groups: hydrolyzable tannins and condensed tannins [57]. Gallotannins are the simplest hydrolyzable tannins. They contain gallic acid substituents esterified with a polyol residue, primarily D-glucose [58]. Condensed tannins are the most abundant polyphenols derived from plants. Condensed tannins are polymeric phenolic compounds made of catechin units and yield anthocyanidin when depolymerized, and are commonly known as proanthocyanidins [49]. Tannins exhibit various pharmacological effects, such as antioxidant and free radical scavenging activities, antimicrobial, anti-cancer, anti-nutritional, and cardio-protective properties [58].

Stilbenes are polyphenolic compounds with a C6-C2-C6 structure that are derived from the secondary metabolism of plants [59]. The most well-known stilbenoid is resveratrol, but there are other interesting compounds like astringin and isorhapontin that are derived from forest biomass and could potentially serve as starting materials for new products [60]. Resveratrol has antioxidant activity that affects the cardiovascular system [61]. Stilbenes have been successfully isolated from the bark of Norway Spruce roots [60] and the grape cane of *Vitis vinifera* L. [62].

Lignans are dimers of phenylpropanoid units linked by the central carbons of their side chains [63]. Lignans are widely distributed throughout the plant kingdom and can be found in various plant parts, including flowers, fruits, roots, rhizomes, leaves, seeds, stems, xylem, and resins [64]. Lignans have various pharmacological activities such as antioxidant [65], and anti-inflammation [66], and can treat breast cancer [67]. 

## 3. Isolation and Purification Method

The extraction of phenolic compounds from natural products has been conducted with several methods, such as maceration [68], percolation [69], Soxhlet extraction [68], reflux extraction [69], decoction [69], ultrasound-assisted extraction (UAE) [70], pulsed electric filed extraction (PEF) [71], enzyme-assisted extraction (EAE) [72], microwave-assisted extraction (MAE) [73], homogenate-assisted extraction (HAE) [74], and subcritical water extraction (SWE) [75]. An extract from plant material is a complex mixture containing different types of natural compounds with different polarities. Therefore, further separation and purification are required to obtain pure bioactive compounds [36]. Several methods have been used to isolate or purify the phenolic compounds that have antioxidant activity. HPLC, MPLC, CCC, HILIC, SFC, column chromatography, and MITs were the methods used for separating active compounds from natural products, with new improvements from 2017 to 2023. In addition, molecularly imprinted polymers also have been developed to isolate compounds with antioxidant activity. 

### 3.1. Medium Pressure Liquid Chromatography

MPLC is one of a wide variety of preparative column chromatography techniques. Separation under pressure allows the use of smaller particle-size supports, increasing the variety of stationary phases that can be used [76]*.* The instrumentation of MPLC is present in Supporting Information Figure S2, which consists of a pump for the mobile phase, a sample injection system, and a self-packed stationary phase (column). Compound separation can be followed automatically by detectors and recorders connected to the outlet of the column or monitored manually by thin-layer chromatography, then collected via a fraction collector [76]*.*

Although MPLC can be used to isolate a compound [77], it is generally used to enrich bioactive compounds (secondary metabolites) from natural products before further purification because of its low cost, high sample load, and high throughput [78]. Therefore, MPLC can be used to remove non-target compounds. The operation pressure used in MPLC is 75–300 psi [79]. 

The MPLC system can be applied to adsorption, partition, affinity, or ion-exchange chromatography [80]. The materials used in the stationary phase include silica gel, MCI GEL^®^ CHP20P, and Sephadex LH-20 [81,82,83]. The choice of column type is critical in MPLC because it can affect the purity and yield. One of the MPLC developments is the use of polyamide columns and MCI GEL^®^ CHP20P. Polyamide column chromatography is a widely used method for separating polyphenols. Polyamide has the ability to adsorb anions through electrostatic interactions [84]. MCI GEL^®^ CHP20P is an adsorption resin that consists of a reversed-phase resin bound to a polystyrene matrix, offering excellent hydrophobicity and efficient separation for polar compounds [39]. The application of polyamide coupled with MCI GEL^®^ CHP20P in MPLC was carried out by Dang et al. [39] to purify bergenin from *Saxifraga atrata* extract. Bergenin is a compound that is derived from trihydroxybenzoic acid (phenolic acid) and has a glycoside attached to it [85]. In the first step, the bergenin fraction was concentrated on a polyamide column (15 × 460 mm) and eluted using a water/acetonitrile (ACN) gradient system. Then, the desired fractions were purified in the second step using MCI GEL^®^ CHP20P (15 × 460 mm). The elution used water/ACN in isocratic mode (5% ACN, 60 min). The authors obtained 1.2 g of bergenin from 180 g of *S. atrata* dry plant material. After purification using MCI GEL^®^ CHP20P, they obtained 714.2 mg of bergenin with >99% purity [39]. Other methods for isolating the bergenin are listed in Table 1. 

Based on Table 1, when viewed based on the percentage yield and purity, the polyamide column coupled with MCI GEL^®^ CHP20P is quite promising, but this method requires instruments during the isolation process. The vacuum liquid chromatography column method could also be chosen: it has a good yield, but the purity is unknown because the authors did not mention it in the article.

Sometimes MPLC is combined with HPLC or preparative HPLC. In this case, MPLC was used for pre-treatment to enrich bioactive compounds in the extract, while HPLC was used to screen and purify the compound. Dawa et al. [89] were the first to combine MPLC and HPLC coupled with online HPLC-1,1-diphenyl-2-picrylhydrazyl (DPPH) detection. They isolated DPPH inhibitors from *S. atrata*. They pre-treated the extract with MPLC using MCI GEL^®^ CHP20P (49 × 460 mm) as a stationary phase and eluted with a mixture of methanol and water, yielding 1.4 g of the target DPPH inhibitors (11.9% recovery). Then, the authors purified the compounds by using HPLC with RP-C18 followed by HILIC column separation. They obtained four phenolic compounds, ethyl gallate, 11-O-galloylbergenin, rutin, and isoquercitrin with >95% purity [89]. 

Four DPPH inhibitors have been also isolated from the methanol extract of *Saxifraga sinomontana*, including 3-methoxy-4-hydroxyphenol-(60-O-galloyl)-1-O-β-D-glucopyranoside (1), 3,4,5-trimethoxyphenyl-(60-O-galloyl)-1-O-β-D-glucopyranoside (2), Saximonsin A (3), and Saximonsin B (4). Compounds 1 and 2 were phenylpropanoid glycoside, while compounds 3 and 4 were phenolic acid. They exhibited potent antioxidant activity with IC_50_ values of 39.6 mM, 46.9 mM, 11.4 mM, and 20.6 mM, respectively [90]. The studies that have applied MPLC to isolate the phenolic antioxidant compounds are described in Table 2.

### 3.2. High Performance Liquid Chromatography

HPLC is a separation and purification method used to obtain compounds with a high purity. One application is the isolation of phenolic compounds. The recycle-HPLC has advantages in separating and isolating compounds, such as increased throughput, shorter separation times, reduced solvent consumption, and full compound recovery [91]. The applications of HPLC in separating or purifying phenolic compounds are listed in Table 3. 

Reverse-phase HPLC (RP-HPLC) is the most commonly used HPLC mode; the stationary phase is less polar than the eluting solvent. A common RP-HPLC stationary phase is surface-modified silica. Usually, modifications were made using RMe_2_SiCl, where R is a linear alkyl group [92]. The eluent used in RP-HPLC is usually a mixture of water and a miscible organic solvent (ACN, methanol, or tetrahydrofuran [THF]) [93]. Semi-preparative RP-HPLC has been used to improve the purification of the isolated compound. Rutin is widely recognized as a powerful natural antioxidant that effectively reduces oxidative stress [94]. Rutin is a glycoside of quercetin, consisting of glucose and rhamnose sugars attached to position C-3 hydroxy group [95]. Yingyue et al. [96] used Sephadex column chromatography and semi-preparative RP-HPLC to purify rutin after extraction from banana leaves. After the liquid–liquid extraction process, the authors purified the fraction by using a Sephadex column. As a result, the purity of rutin was 74–84%. Then, they further purified rutin by using semi-preparative RP-HPLC. The final rutin was 98.4% pure. The scheme of this method can be seen in Supporting Information Figure S3 (isolation process 1). Based on these results, semi-preparative RP-HPLC can enhance the purity of isolated compounds [96]. Rutin has also been isolated from *S. atrata* by using a combination of MPLC and HPLC coupled with online HPLC-DPPH detection [89]. The scheme is shown as isolation process 2 in Supporting Information Figure S3. Both methods 1 and 2 produce products with high purity (>95%); however, method 1 is more effective and easier to operate because it only requires HPLC instruments. Method 2 can be helpful when the compound isolated is only a DPPH inhibitor (such as rutin), so the isolation focuses on compounds with DPPH inhibitory activity.

Recycle HPLC has been developed to increase the phenolic separation efficiency of the process while keeping the peak dispersion as low as possible. This goal is achieved by installing a recycle valve on the HPLC system (at the preparative or semi-preparative scale) to return the unresolved peaks to the column (Supporting Information Figure S4). No new solvent is required during the recycling period, which is another advantage of this method [97]. Recycle HPLC is an effective method for separating compounds that elute close together, and the system’s ability to remove contaminants between chromatography cycles ensures the high purity of the isolated compounds [98]. Molo et al. [38] used C18, GS-320 columns to purify phenolic compounds from a subfraction of *Chaerophyllum bulbosum* extract. For the C18 column, they purified luteolin-7-*O*-β-D-glucopyranoside, a flavonoid compound, with methanol and water (50:50, v:v) as a mobile phase. GS-320 columns were used to purify quercetin-3-O-β- D-glucopyranoside. Luteolin-7-*O*-β-D-glucopyranoside and quercetin-3-O-β- D-glucopyranoside exhibited higher DPPH^•^ and ABTS^•+^ scavenging activities compared to BHA and α-tocopherol standards [38].

Preparative high-performance/high-pressure liquid chromatography (prep-HPLC) usually implies the use of large columns, large sample loading volumes, and high flow rates on HPLC systems to purify or separate compounds in large volumes [99]. Prep-HPLC was developed to isolate quercetin-3-O-α-L-rhamnopyranosyl-(1 → 6)-β-D-glucopyranoside a quercetin glycosides using a gradient system (15–25% acetonitrile) for 45 min. This compound showed the highest antioxidant activity in DPPH, OH radicals scavenging, and CUPRAC assay with IC_50_ 28.8, 145.8, and 13.9 μM, respectively [100]. The DPPH activity of this compound was higher than quercetin (1.6 times) and ascorbic acid (2.0 times) [100]. 

**Table 3:** Application HPLC to isolate or purify phenolic compounds from natural products.

Sample	Compound	System	Stationary Phase	Mobile Phase	Yield (%) *	Ref.
*Theobroma cacao*	(+)-Catechin, d (−)-epicatechin, B-type dimer of flavan-3-ols, epicatechin, trimer flavan-3-ols	Semi- preparative HPLC	NM	NM	NM	[101]
*Chaerophyllum bulbosum*	Quercetin-3-O-β- D-glucopyranoside	Recycle HPLC	GS-320 column	100% Methanol	0.011	[38]
	Luteolin-7-O β-D-glucopyranoside		C18	50% Methanol/50% water	0.007	
Banana leaves (*Musa balbisiana*)	Rutin	Semi- preparative RP-HPLC for purification	C18	50% Methanol/50% water	3.24	[96]
*Hippocrepis emerus* flowers	quercetin-3-O-α-L-rhamnopyranosyl-(1 → 6)-β-D-glucopyranoside	Prep-HPLC	NM	gradient system (15–25% acetonitrile) for 45 min	0.517	[100]
	quercetin-7-O-α-L-rhamnopyranoside	Semipreparative-HPLC	NM	gradient system (30–35% acetonitrile) fo 20 min)	0.138	
*Smilax glabra* Roxb	(-)-Epicatechin	Preparative HPLC	Waters Sunfire Prep C18 OBDTM 250 × 19 mm, 5 μm	Acetonitrile (A) and water with 0.3% formic acid (B) with gradient elution	1.77	[102]
	Neoastilbin				11.04	
	Astilbin				18.10	
	Neoisoastilbin				4.09	
	Isoastilbin				5.03	
*Pleioblastus amarus* shoots	3-O-feruloylquinic acid	semi-preparative HPLC	C-18	Methanol-0.1% acetic acid (60:40, *v*/*v*)	0.03	[103]
*Frankenia pulverulenta*	Gallic acid	Prep-HPLC	C-18, 5 µm	Gradient system using solvent A (water: 0.1% TFA) and solvent B (ACN/0.1% TFA)	NM	[104]
	Catechin					
	Quercetin					
*Magnolia officinalis*	Syringin	Semi-preparative HPLC	C-18, 5 µm	Gradient system using solvent A (water containing 0.2% acetic acid, *v*/*v*) and B (methanol)	NM	[105]
	Magnoloside B					
	Magnoloside A					
	Magnoloside F					
	Magnolol			isocratic system using solvent A (water containing 0.2% acetic acid, *v*/*v*) and B (methanol), with 80% B		
	Obvatol					
	Honokiol					
*Origanum* *minutiflorum*	Eriodictyol	Semi-preparative HPLC	NM	NM	NM	[106]
	Luteolin					
	Rosmarinic acid					
*Teucrium hyrcanicum* L.	Acteoside	Semi preparative HPLC	NM	Gradient system using water:acetic acid (99:1) (solvent A) and acetonitrile (solvent B)	NM	[107]
*Moringa oleifera* leaves	Isoquercitrin	Semipreparative HPLC	C-18	Gradient system using water (solvent A) and an acetonitrile and water mixture (40:60, *v*/*v*) (solvent B)	0.02	[13]
	Astragalin				0.002	
	3-O-caffeoylquinic acid				0.003	
*Schinopsis brasiliensis*	gallic acid 4-O-b-D-(60-Ogalloyl)-glucopyranoside	Semi-preparative HPLC	NM	Methanol:water (3:7)	0.18	[108]
	2-Hydroxy-4-methoxyphenol 1-O-b-D-(60-O-galloyl) glucopyranoside				0.16	
	4,9-Dihydroxypropiophenone-9-O-(60-O-galloyl)-b-Dglucopyranoside				0.17	
	3,4-di-O-galloyl-quinic acid				0.31	
	4-hydroxy-3-methoxyphenol-1-O-(60-O-galloyl)- b-D-glucopyranoside			Methanol:water (1:3)	0.17	
	4-hydroxy-2- methoxyphenol-1-O-b-D-(60-O-galloyl) glucopyranoside				0.19	
*Artocarpus* *elasticus*	Artonin W	Prep-HPLC	C18	30% Methanol in water	0.092	[109]
	Artorigidinone B				0.088	
	Cycloartobiloxanthone				0.078	
*Berberis baluchistanica*	Pakistanine	Preparative recycling HPLC	C18	Acetonitrile:water (60:40)	0.34	[110]
*Malus prunifolia* (Willd.) Borkh.	Sachaliside	Semi-preparative HPLC	Xbridge^®^ (250 mm × 4.6 mm, 10 μm,)	Methanol–0.1% formic acid-water (15:85)	NM	[111]
	Chlorogenic acid			ACN-0.1% formic acid-water (15:85)		
	Epicatechin			ACN-0.1% formic acid-water (8:92)		
	Procyanidin B2			ACN-0.1% formic acid-water (14:86)		
*Nitraria tangutorum*	Tyrosol 8-O-β-d’glucopyranoside	Prep-HPLC	XCharge 18	5%−55% of acetonitrile with 0.2% formic acid	NM	[112]
	Querceitn 3-O-(2G-rhamnosylrutinoside)					
	Vanillic acid			5%−35% of acetonitrile with 0.2% formic acid		
*Tithonia diversifolia* (Hemsl.) A. Gray	(*E*)-3-(((3-(3,4-dihydroxyphenyl)acryloyl)oxy)methyl)-2-methyloxyrane-2-carboxylic acid	Prep-HPLC for purification	C18	Phosphorous acid 0.05%:isopropyl alcohol (93:7)	0.003	[113]

NM, not mentioned in the article; RP-HPLC, reverse-phase high-performance liquid chromatography. * Yield (%): ratio of isolate mass with crude extract mass.

### 3.3. Counter Current Chromatography

CCC is a form of liquid–liquid partition chromatography that uses two immiscible liquids. One phase is maintained as the stationary phase in the absence of an adsorption matrix. The second phase passes through the stationary phase and is efficiently equilibrated by utilizing a hydrodynamic or turbulent mixture [114]. The difference compared with other chromatographic systems is that this method does not require solid support: the stationary phase is held in the column by gravity or centrifugal force [115].

CCC has also been used to isolate bioactive compounds. Several CCC methods have been developed for isolation, one of which is the use of the pH zone refinement CPC. In addition, a new approach using a DES with HSCCC has been developed in an effort to use a more environmentally friendly solvent for isolation. Here, we will discuss the CCC method with its performance in compound isolation and compare the results of different CCC systems to evaluate the best one.

**Figure 3:**
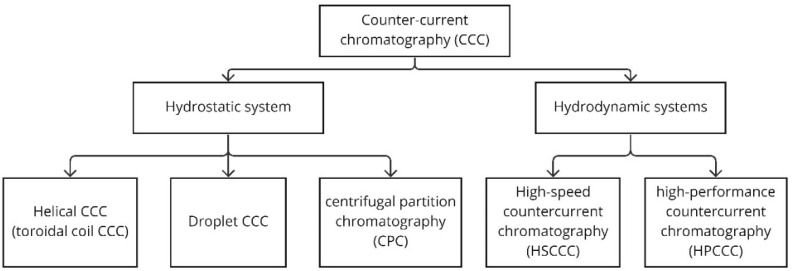
Counter-current chromatography (CCC) systems.

CCC is divided into hydrostatic and hydrodynamic equilibrium systems (Figure 3) [116]. A hydrostatic system uses a stable force field to hold the stationary phase on the column while the mobile phase flows through the column. Helical CCC (toroidal coil CCC), droplet CCC, and CPC are hydrostatic systems. The hydrodynamic systems use the Archimedes screw effect, which promotes the constant mixing of the two phases while maintaining one of the phases as the stationary phase [116]. HSCCC and HPCCC are hydrodynamic systems.

CPC is widely used to purify natural products by partitioning a sample between two immiscible phases. Several compounds have been isolated using this method, one of which is the flavonoid in *Bryophyllum pinnatum* (Lam.) Oken (Crassulaceae) [40], and phenolic compounds from *Anogeissus leiocarpus* Guill. and Perr. (Combretaceae) [117]. 

As mentioned above, CPC is a form of hydrostatic CCC [118] in which a stationary liquid phase is supplied to the rotor while it rotates at a moderate rotational speed and is maintained in the rotor by the resulting centrifugal force. Then, the mobile phase, which contains the solute to be extracted is delivered under pressure to the rotor and pumped through the stationary phase [119]. The partition coefficient (Kd) indicates the separation of constituents, which is determined by the concentration of the target compound in the stationary phase divided by the concentration in the mobile phase. Therefore, components with a high affinity for the mobile phase elute early, and components with a high affinity for the stationary phase elute later [120,121,122,123]. The advantages of CPC over the droplet CCC (DCCC) were a faster movement of the mobile phase past the stationary phase than DCCC because CPC generates a centrifugal force of the rotor, a greater flow rate, and a more efficient method [124]. CPC has been applied to isolate three flavonols from the bark of *Weinmannia trichosperma* Cav., such as isoastilbin, neoisoastilbin, and neoastilbin using the HEMWAT system (hexane-ethyl acetate-methanol-water; ratio 1:9:1:9) [125]. These compounds exhibited potent antioxidant activity not only in DPPH and the 3-ethylbenzothiazoline-6-sulfonic acid (ABTS) radical scavenging but also in the ferric-reducing ability of the plasma (FRAP) system [102]. 

HSCCC uses hydrodynamic equilibrium. The instrument consists of a spindle, a planetary axis, and a couple of rotating axes that use a hydrodynamic device where separation occurs in a multilayer coil. It consists of a long piece of endless tubing wrapped in multiple layers around a holder. Several of the resulting coils can be connected in series to increase the total volume of the instrument. The coil is subjected to a centrifugal force field and rotates around its axis while at the same time rotating around the central axis of the system. This motion results in the retention of the stationary phase and partition of the analyte between the two liquid phases [61,68].

As with forms of CCC, the choice of the solvent system is critical in this method. The solvent system often consists of organic solvents, including *n*-hexane, *n*-butanol, dichloromethane, methanol, and ethyl acetate. However, these solvents are dangerous for researchers and the environment [126,127,128]. A DES for HSCCC has been developed to overcome this drawback. The combination of DES and HSCCC is recent in the development of methods to isolate bioactive compounds from natural products. DES, composed of hydrogen bond donors and acceptors, are a new generation of room-temperature liquid salts that have been widely used to extract bioactive compounds [129,130,131]. Cai et al. [41] developed an HSCCC system using DES to extract and separate flavonoids from *Malus hupehensis*. Based on their optimization experiment, they chose choline chloride/glucose, water, and ethyl acetate (1:1:2, *v*/*v*) for HSCCC separation. They successfully isolated three phenolic compounds with this method, namely avicularin, phloridzin, and sieboldin, each with >92% purity. Overall, the authors demonstrated that two HSCCC separations with DES are a valuable and environmentally friendly way to separate pure compounds from extracts [41]. In another study, avicularin, phloridzin, and sieboldin were identified as antioxidant compounds [132,133]. The activity of avicularin was determined using DPPH and hydroxyl (OH) scavenging assays. At a concentration of 100 mg/L, avicularin exhibited effective antioxidant activity, with OH radical scavenging rates reaching 87.54% [132]. The antioxidant capacity of sieboldin was demonstrated by its ability to prevent vasoconstriction and inhibit advanced glycation end-products (AGEs) formation [133].

HSCCC was also successful in separating and purifying the antioxidant phenolic glycoside in an extract of *Castanopsis chinensis* Hance. In this study, preliminary separation by multistep column chromatography was applied to obtain the phenolic glycoside fraction that contains the mixture of compounds. The HSCCC successfully separated two antioxidant phenolic compounds with higher purity, chinensin D (93.0%) and chinensin E (95.7%) [134]. 

HPCCC works much the same way as HSCCC. Separation using HSCCC is characterized by long separation times, typically 3–8 h. So, HPCCC instruments have been developed to enable high-resolution separations with 20–60 min of elution. It is achieved by providing columns that maintain >75% steady-state retention at semi-preparative mobile phase flow rates of ≥6 mL/min. Flow rates of 20–100 mL/min are used for preparative-scale separations, maintaining >75% stationary phase retention [135]. The studies for separating phenolic compounds with antioxidant activity using HSCCC and HPCCC are presented in Table 4.

In general, the CCC systems have advantages compared with HPLC and MPLC: they do not require a solid column, there is low solvent consumption, there is no irreversible loss of sample because chemosorption can be avoided, there is higher sample recovery, and a high load capacity [147,148,149]. However, CCC systems require time, especially to optimize the solvent system, and instability of the solvent system may occur.

### 3.4. Hydrophilic Interaction Liquid Chromatography 

Hydrophilic Interaction Liquid Chromatography or HILIC is a type of partition chromatography that uses a polar stationary phase. The partition occurs between a non-polar or organic region in the mobile phase and a polar water-enriched layer at the surface of the polar stationary phase [150]. Several stationary phases, including silanol-derivatized phases such as amino-, amide-, cyanopropyl-, carbamate-, diol-, and polyol-, have been developed for HILIC [151]. HILIC has successfully applied for the enrichment of the compound in the extract in isolation. In isolation, HILIC is usually combined with RPLC, often called two-dimensional liquid chromatography (2D-LC). 2D-LC is widely used for separating complex samples due to its high resolution and large peak capacity [152]. 

Dang et al. (2018) have isolated the antioxidant phenolic compound from *Dracocephalum heterophyllum* using an offline two-dimensional reversed-phase/hydrophilic interaction liquid chromatography (2D RP/HILIC) technique guided by on-line HPLC-DPPH [153]. During the isolation process, the C18 preparative column was used for first-dimensional (1D) separation which resulted in six antioxidative fractions with a recovery rate of 61.4% out of the ethyl acetate fraction. For the second-dimensional (2D) separation, a HILIC XAmide preparative column was used. A total of eight antioxidants (caffeoyl-β-D glucopyranoside, ferruginoside B, verbascoside, 2′-O-acetylplantamajoside, sibiricin A, luteolin, rosmarinic acid, and methyl rosmarinate) were isolated from *D. heterophyllum*, with a purity of over 95% [153]. The application of separation using HILIC in the isolation process can be seen in Table 5. 

### 3.5. Column Chromatography 

Column chromatography is used to separate impurities and purify biological mixtures. It is also used to isolate active molecules and extract metabolites from various samples [157]. The solid and liquid samples can be separated and purified by this method. The stationary phase of column chromatography is placed inside a narrow tube (column), and the stationary adsorbed and separated passing compounds with the help of a liquid mobile phase. Due to their chemical nature, compounds are adsorbed, and elution is based on the differential adsorption of substances by the stationary phase [158]. The studies that used column chromatography for phenolic compound isolation can be seen in Table 6. 

#### 3.5.1. Silica Gel Chromatography 

Silica gel has a silanol group, and it is a polar absorbent. Molecules are held in silica gel by hydrogen bonding and dipole-dipole interactions. For example, polar natural substances are retained longer on silica gel columns than non-polar ones [159]. Pyrogallol, rutin, and morin are phenolic compounds isolated from the ethyl acetate fraction of *Bergenia ciliata* by silica gel column chromatography. Solvent systems, including ethyl acetate and n-hexane were employed, with polarities ranging from 1% to 50%. Pyrogallol, rutin, and morin have been demonstrated to be effective against free radicals ABTS and DPPH. Notably, pyrogallol has exhibited the highest efficacy among them [160].

Isolation using silica gel column chromatography is still used because it boasts several advantages, including its ease of use; the stationary phase is stable and does not readily decompose [30]. However, the drawback of this method is that it is time-consuming and requires a large amount of solvent. 

#### 3.5.2. Size Exclusion Chromatography 

Size-exclusion chromatography is a type of partition chromatography, used to separate molecules based on their sizes. Sephadex^®^LH-20 is a stationary phase in size exclusion chromatography widely utilized for isolating the bioactive compounds in natural products. Sephadex^®^LH-20 is a size exclusion column prepared by hydroxypropylated dextran gel [161] that has both hydrophilic and lipophilic properties, and is stable in all solvents except strong acid, and contains strong oxidizing agents [162]. Naringin belongs to the flavonoid class known as flavanones that act as antioxidant and anticancer [163]. Naringin has been successfully isolated from pomelo peel extract using Sephadex^®^LH-20 with higher purity (95.7 ± 0.23%). Other phenolic compounds have also been successfully separated with Sephadex^®^LH-20, including quercetin and (2R)-eriodictyol in mulberry fruit extract [164]. The advantage of using Sephadex^®^LH-20 for isolating is that it allows for separating a wide range of natural products using either aqueous or non-aqueous solvents [159]. The disadvantage of using Sephadex^®^LH-20 is that it requires choosing the right solvent because the particle size and exclusion limit differ depending on the solvent used for swelling.

**Table 6:** A list of studies that used column chromatography.

Sample	Compound	Type of Sorbent	Mobile Phase	Yield (%) *	Ref.
*Pistacia integerrima* gall	Quercetin and pyrogallol	Silica gel	Mixture of ethyl acetate: n-hexane with different concentrations (1–60%)	NM	[37]
Rhizomes of the *Bergenia ciliata*	pyrogallol, rutin and morin	Silica gel	*n*-hexane at the first and followed by increase in polarity of *n*-Hexane/ethyl acetate gradients up to 50% ethyl acetate/n-hexane (1:1) gradient	NM	[160]
*Endopleura uchi*	Bergenin	silica gel LC60A (70–200 μm)	Chloroform/ethanol (7:3) isocratic	5.4% (Leave extract), 5.73% (Twigs extract), and 6.09% (Bark extract)	[165]
*Alseodaphne semecarpifolia* Nees	Icariin	Silica gel	Gradient elution: n-hexane: ethyl acetate (100:0 → 0:100), then ethyl acetate: petroleum ether (100:0 → 0:100), then petroleum ether: chloroform (100:0 → 0:100)	1.34	[166]
	Baicalein			1.23	
*Litsea glaucescens*	Epicatechin	Silica gel 60 column (100 cm × 5 cm)	Gradient elution using mixture of hexane-ethyl acetate-methanol mixtures	NM	[167]
	Quercitrin				
	Kaempferol				
*Boesenbergia rotunda*	2′, 4′-dihydroxy-6-methoxychalcone	Silica gel	Hexane-ethyl acetate (6:4)	0.125	[168]
	5-hydroxy-7-methoxyflavanone			0.35	
	5, 7-dihydroxyflavanone			0.2	
*Apocynum venetum* tea	(−)-epicatechin	Silica gel	Hexane	0.003	[169]
*Jatropha podagrica*	Fraxetin	Silica gel	Hexane and ethyl acetate at 8:2, 7:3, and 6:4 ratios,	0.059	[170]
*Euphorbia balsamifera*	Quercetin-3-O-glucopyranoside	Silica gel	Methanol	0.003	[171]
	Isoorientin	Silica gel	chloroform/methanol (7:3)	0.004	
*Hibiscus rosa sinensis*	Hibiscetin-3-glucoside	Silica Gel G-60	NM	NM	[33]
*Cordia sebestena* flower	Hesperitin	Silica gel	Chloroform/methanol (60:40)	NM	[172]
*Ipomoea* *pes-caprea* (Convolvulaceae) leaves	(5,7-dihydroxy-4-phenyl-2H-chromen-2- one)	Silica gel	100% hexane followed by a gradient mixture of hexane: methanol (95:5–100).	1.02	[173]
*Afzelia africana*	3,3′ -di-O-methyl ellagic acid.	Silica gel	Petroleum ether-ethyl acetate (85:15)	0.029	[174]
*Zygophyllum simplex* L.	Myricitrin	Silica gel 100 C18	Methanol:water (1:9)	0.105	[175]
	Luteolin-7- O-β-D-glucoside		Methanol:water (2:8)	0.084	
*Calendula tripterocarpa* Rupr	Quercetin	Silica gel	Ethyl acetate-methanol–water (90:5:4)	0.32	[176]
	Scopoletin			0.23	
*Ferulago cassia*	Peucedanol	Silica gel	Hexane:ethyl acetate (76:24)	0.1	[177]
	Umbelliferone		Hexane:ethyl acetate (63:35)	0.16	
*Perilla frutescens* (L.) Britt.	Ferulic acid	Silica gel	Mixture of chloroform and methanol	0.042	[178]
	Luteolin	Silica gel	Mixture of chloroform and methanol	0.033	
	Apigenin	Silica gel	Chloroform:methanol mixture (12:1 to 4:1)	0.042	
	Caffeic acid	Combination of silica gel and Sephadex LH-20	NM	0.024	
	Rosmarinic acid	Sephadex LH-20	90% of methanol	0.16	
*Retama monosperma* (L.) Boiss.	Quercetin	Silica gel	Mixture of Hexane, diethyl ether, and ethyl acetate with gradient elution	NM	[179]
	6-methoxykaempferol				
	Kaempferol				
*Origanum rotundifolium*	Apigenin	Silica gel	Solvent system with increasing polarity from hexane to ethyl acetate and ethyl acetate- methanol	NM	[180]
	Ferulic acid				
	Vitexin				
	Rosmarinic acid				
	Globoidnan A				
Palmyra palm (*Borassus flabellifer* Linn.) syrup	2,3,4-trihydroxy-5 methylacetophenone	Silica gel	Mixture of dichloromethane and methanol	1.82	[181]
*Prunus mahaleb* L.	Gallic acid	Silica gel	Mixture of chloroform and methanol with different ratio	0.0067	[182]
*Odontites serotina* (Lam.) Dum	Acteoside	Silica gel	NM	NM	[183]
*Euphorbia geniculata*	Gallic acid	Sephadex LH-20	20% of methanol in water	NM	[184]
	Ellagic acid				
	Rutin		40% of methanol in water		
	Quercetin		100% of methanol		
Pomelo peels	Naringin	Sephadex LH-20	30% of ethanol	NM	[185]
*Desmodium caudatum*	Descaudatine A	Combination of silica gel, Sephadex LH-20 and C-18	NM	NM	[186]
	8-Dimethylallyltaxifolin				
*Arisaema heterophyllum* tubers	6,7-dihydroxy-2-(4-hydroxyphenyl)-4 H-chromen-4-one	Sephadex LH-20	Methanol	0.047	[187]
	(E)-4-(3-hydroxypropyl-1-en-1-yl)phenol			0.005	
Mulberry fruit (*Morus alba* L.)	(2R)-eriodictyol	Sephadex LH-20	Mixture of methanol/water, 50:50 to 70:30 (*v*/*v*)	0.006	[164]
	Quercetin			0.0004	
*Ombrophytum subterraneum* (Aspl.) B. Hansen (Balanophoraceae)	3′,5,5′,7-tetrahydroxyflavanone 7-O-β-D-1 → 6 diglucoside	Sephadex LH-20	Mixture of methanol/water, (8:2)	17.82	[188]
Mulberry leaves	Rutin	Sephadex LH-20	Methanol:water (3:7)	0.041	[189]
	Isoquercetin		Methanol:water (1:9)	0.039	
*Manilkara hexandra* fruit	Gallic acid	Sephadex LH-20	n-Butanol–Isopropyl alcohol–Water	0.133	[190]
	Taxifolin			0.066	
	Myricetin		10% MeOH and n-Butanol–Isopropyl alcohol–Water	0.133	
	Quercetin			0.133	

NM, not mentioned in the article. * Yield (%): ratio of isolate mass and crude extract mass.

### 3.6. Supercritical Fluid Chromatography (SFC)

Supercritical fluid chromatography (SFC) is a separation method that uses compressed gas above the critical temperature/pressure instead of organic solvent [191]. SFC is a green separation technique that uses mostly supercritical carbon dioxide as the mobile phase, with the eluting power controlled by the addition of organic solvent as an organic modifier. This method is known for its low operational cost and is considered an environmentally friendly alternative [192]. SFC is the preferred method for separating natural products on an analytical or preparative scale (Table 7). This method has been applied to separate curcumin, demethoxycurcumin, and bisdemethoxycurcumin directly from turmeric on a preparative scale. After a single step of supercritical fluid chromatography separation, 20.8 mg of curcumin (97.9% purity), 7.0 mg of demethoxycurcumin (91.1%), and 4.6 mg of bisdemethoxycurcumin (94.8%) were obtained with a mean recovery of 76.6% [193].

A two-dimensional offline SFC/RPLC system was developed to separate and prepare lignans from *Fructus Arctii*. SFC was utilized in the first dimension to prepare the lignin fractions, while RPLC was employed in the second dimension to produce high-purity lignin compounds. This method has advantages such as high loading ability, and short time analysis [194]. 

**Table 7:** A list of studies that used the SFC.

Sample	Compound	Mode Separation	Purity	Yield (%) *	Ref.
*Fructus Arctii*	Matairesinol	2D-SFC/RPLC	>90%	NM	[194]
	Arctigenin				
	Lappaol C				
Fructus Cnidii	Osthole and	Semi-preparative SFC	98.9%	19.6	[195]
	Imperatorin		98.2%	24.4	
*Alpinia officinarum*	Pinocembrin	SFC/preparative SFC	99.9%	NM	[196]
	Galangin		99.5%		
	Kaempferide		98.5%		

* Yield (%): ratio of isolate mass and crude extract mass.

### 3.7. Molecularly Imprinted Technology

MITs provide a versatile, tailor-made technique to separate and purify specific target molecules. MIPs are synthetic polymers with excellent properties due to their low cost, ease of fabrication, high selectivity, and good reusability [197]. MIPs have three-dimensional (3D) structures. They are synthesized by copolymerizing functional monomers and crosslinkers in the presence of template molecules [198,199]. The template molecules are then removed to obtain a polyporous polymer with complementary cavities to the shape, size, and functional groups of the template molecule [200,201]. Despite many methods existing for MIP synthesis, only several methods have been developed to synthesize MIPs for separating or isolating phenolic compounds in extracts, including in situ polymerization [202,203], Pickering emulsion polymerization [204], bulk polymerization [205,206], precipitation polymerization [207,208], and the surface molecular imprinting technique (SMIT) [209,210]. 

Bulk polymerization is conventionally used for MIP synthesis. The template molecules, functional monomer, crosslinker, and initiator are mixed in a non-polar solvent in specific ratios. Polymerization is initiated by light or thermal irradiation. The solid polymers must be ground and sieved, and then template molecules are removed from the polymer to obtain specific cavities [211]. Precipitation polymerization is a simple and popular method to produce the MIP. This method can make high-quality monodisperse MIP microspheres without stabilizers or emulsifiers. Unlike bulk polymerization, grinding is not carried out because it could damage a specific surface area or imprinting site, thus reducing the adsorption capacity [212]. MIPs produced using this method have uniform shapes and sizes [213]. The surface molecularly imprinted technique (SMIT) is a polymerization method that occurs on the surface of solid matrixes. The binding sites are distributed on the outer layer of the surface of the solid matrixes. One of the solid matrixes used in this method is magnetic Fe_3_O_4_ [214]; it is called magnetic molecularly imprinted polymer (MMIP). In contrast to the previously mentioned techniques, SMIT has advantages such as faster binding kinetic, higher separation efficiency, and minimizing the embedded phenomenon [215].

**Figure 4:**
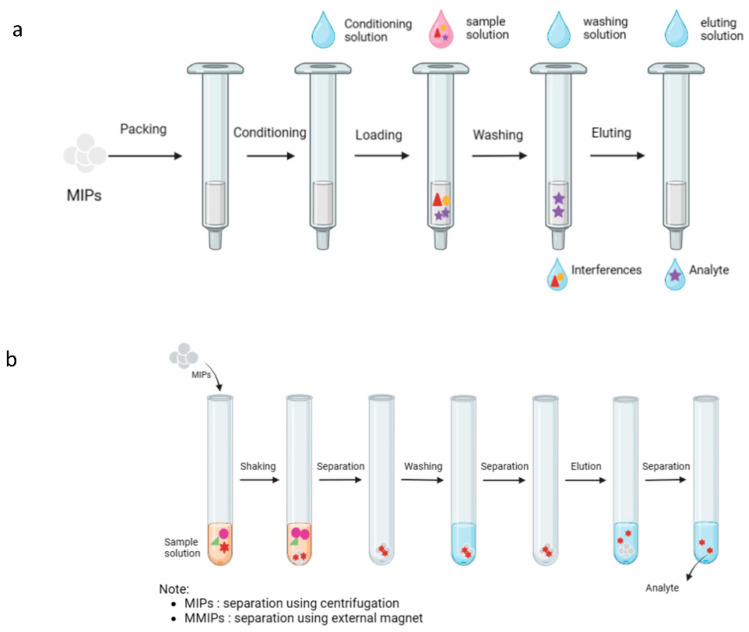
Schemes of separation using (**a**) solid-phase extraction (SPE) and (**b**) dispersive solid-phase extraction (DSPE).

MIPs have mostly been used in sample pre-treatment as a sorbent for solid-phase extraction (MISPE) and dispersive solid-phase extraction (MIDSPE). In MISPE, the MIP is packed in an SPE cartridge. The process includes conditioning MISPE, loading the sample, washing to clear interference, and eluting the analyte [216]. In MIDSPE, the MIP is directly applied to the liquid volume of the sample solution. The entire procedure involves shaking and centrifugation for separation. There are two types of sorbents used in MIDSPE, namely non-magnetic MIP and MMIP [217]. An external magnet can be used in the separation process when MMIP is applied as the MIDSPE sorbent [218]. Figure 4 shows the schemes for MISPE and MIDSPE. In addition, MIPs can be used for chromatographic separation (monolithic column). Monolithic column MIP can be prepared directly by in situ free-radical polymerization within the chromatographic column. A grinding, sieving, and packing column are not needed in this stage [219]. The application of MIPs for extraction, separation, or purification are listed in Table 8. 

Hosny et al. [205] synthesized MIPs using bulk polymerization to isolate a phenolic compound which is sinapic acid from broccoli. This phenolic compound has tremendous antioxidant potential [220] and could be used to treat several pathologies, including diabetes [221], infection [222], inflammation [223], and cancer [224]. The authors determined the optimum MIP for isolating sinapic acid, synthesized using 1:4:20 as the molar ratio of template to the functional monomer (4-vinyl pyridine) to the crosslinker (ethylene glycol dimethacrylate [EGDMA]). 4-Vinyl pyridine acts as a hydrogen bond acceptor to form hydrogen bonding with sinapic acid and its role by π–π stacking [205]. The authors used dimethyl sulfoxide (DMSO) as a porogen because of its low polarity, which avoids hydrogen bonding with template molecules [225]. They determined the binding isotherm by incubating 15 mg of MIP with 2 mL of different concentrations of sinapic acid prepared in pure water (1 × 10^−2^ to 2.5 × 10^−4^ M) for 2 h. They calculated the amount of template molecule bound and constructed a Scatchard plot, which showed two linear regions based on the ratio of bound to free template (Q/F) against the amount of bound template (Q). This is because the MIP has heterogenous binding sites [205]. The maximum number of binding sites available for binding MIP (Q_max_) was 117.51 µmol/g for the regions of high-affinity areas and 572.098 µmol/g for the low-affinity areas. They evaluated the MIP with the total extract and the ethyl acetate fraction of *Botrytis italica* L. (broccoli) by using HPLC. The total extract contained sinapic acid and caffeic acid, while the ethyl acetate fraction contained sinapic acid, ferulic acid, and caffeic acid (the latter two are analogues of sinapic acid). The authors also evaluated the selectivity of their synthesized MIP. After binding between the total extract with MIP, the peak area of sinapic acid decreased by 90% while the peak area of caffeic acid decreased by 65%. Furthermore, after binding between the ethyl acetate fraction and the MIP, the chromatogram showed no peak for sinapic acid, the peak area of ferulic acid had decreased by 60%, and the peak area of caffeic acid had decreased by 11%. The authors concluded that the MIP has an excellent binding affinity and good selectivity toward sinapic acid [205].

**Table 8:** The application of molecularly imprinted polymers (MIPs) for extraction, separation, or purification of bioactive compounds from natural products.

Synthesis Method	Sample	Compound	Sample Pre-Treatment Method	Adsorption Capacity	Yield (%)	Ref.
Bulk polymerization	*Rosmarinus officinalis* L	Rosmarinic acid	MISPE	15.49 mg/g	49.11 ± 4.58 mg/g	[43]
	*Rhodiola crenulata*	Salidroside	MISPE	28.13 mg/g	NM	[226]
	green coffee bean extract	Caffeic acid	MISPE	1.03 mg/g	42%	[227]
		Chlorogenic acid		NM	49%	
Pickering emulsion polymerization	Spina Gleditsiae	Quercetin	NM	0.521 mg/g	NM	[204]
Precipitation polymerization	*Carthamus tinctorius* L. and *Abelmoschus manihot* (Linn.)	Myricetin	MISPE	11.80 mg/g	79.82–83.91% and 81.50–84.32%,	[228]
	*Salvia officinalis* leaves	Rosmarinic acid	UA-DSPE	NM	77.80%	[229]
Surface molecular imprinting	*Citrus reticulata* Blanco	Hesperetin	MIDSPE with MMIP	7.316 mg/g	NM	[209]
	Apple sample	Kaempferol	MIDSPE with MMIP	3.84 mg/g	NM	[230]
	Spiked sample in *Larix griffithiana*	Dihydroquercetin	MIDSPE with MMIP	77.72 ± 3.56 mg/g	NM	[231]
	*Polygonum cuspidatum.*	Resveratrol	MIDSPE	11.56 mg/g	23.74%	[232]

Note: MMIP, magnetic molecularly imprinted polymer; MIDSPE, molecularly imprinted dispersive solid-phase extraction; MISPE, molecularly imprinted solid-phase extraction; UA-DSPE, ultrasonic-assisted dispersive solid phase extraction; NM, not mentioned in the article.

Wang et al. [209] synthesized MMIP for the selective separation of hesperetin from the dried pericarp of *Citrus reticulata* Blanco. Hesperetin is a flavanone derivate that has myriad pharmacological activities such as anti-cancer [233], anti-inflammation [234], and anti-hyperglycemia [235]. They added 40 mg of MMIP into 2.5 mL of extract solution and then shook the mixture at 30 °C for 2 h to separate hesperetin. They separated the MMIP by using an external magnetic field. They used a mixture of methanol and acetic acid (9:1) to wash the MMIP for 2 h. The eluent contained hesperetin was evaporated and dissolved. The solution was then analyzed by HPLC. They found that the MMIP effectively and selectively separates hesperetin from the dried pericarp extract of *C. reticulata* and highlighted that it could be used for the rapid enrichment and isolation of hesperetin from other natural plants [209].

Several special strategies have been developed to improve the analytical performance of MIPs and to increase the efficiency of the separation process. A dummy template is used to avoid template leakage when using a target analyte as a template molecule. Dummy templates have an analogous structure to the target analyte molecules, with similar shape, size, and functional group, but they do not interfere with analytical determinations [236,237]. Eidi et al. [86] developed a dummy template molecularly imprinted polymer (DMIP) for the selective isolation of sesquiterpene coumarins from asafoetida. They used 7-hydroxycomarin (7-HC), the parent compound of sesquiterpene coumarins, as a dummy template. Bulk polymerization was used to synthesize the DMIP. The optimized polymer was synthesized using methacrylic acid (MMA) as a functional monomer with a 1:6 molar ratio of dummy template to functional monomer. The DMIP was used as a sorbent for SPE (DMISPE). After extraction using DMISPE, the peak area in the chromatogram of asafoetida extract was on average three times more than that without DMISPE. These results show that DMISPE was efficient for selective extraction and clean-up of the sesquiterpene coumarin from the asafoetida extract [206].

Overall, MITs have advantages for separating bioactive compounds: a rapid and easy procedure, selective isolation, fewer impurities, and recoveries of isolates. In contrast, this method’s drawbacks are that the mass of the isolate depends on the binding capacity of the MIP sorbent and requires the additional isolation method when using MIP multi-templates. 

### 3.8. High-Performance Thin Layer Chromatography

HPTLC is an enhanced version of TLC (Thin Layer Chromatography). HPTLC employs various techniques to achieve better separation and analysis of compounds. For instance, HPTLC uses TLC plates with finer particle sizes in the stationary phase, resulting in better resolution [238]. The HPTLC cannot only be applied for separating or isolating the active compounds [238,239], but can also applied for the estimation of the isolated compound [240]. Jug et al. (2021) used the offline multidimensional HPTLC for fractionation and isolation of flavan-3-ols, proanthocyanidins, and anthraquinones derivates from Japanese knotweed rhizome bark extract. A combination of stationary plates was used in this study. Preparative TLC silica gel was used in first-dimension fractionation, HPTLC cellulose plate and HPTLC silica gel were used in second-dimension fractionation, and the HPTLC silica gel was used in third-dimension fractionation. In the isolation process, post-chromatographic derivatization was used to identify the analyte target using 4-dimethylaminocinnamaldehyde (DMACA) to distinguish the flavan-3-ols and proanthocyanidins derivates from other compounds. HPTLC-MS was also used to characterize the isolated compounds. In this study, HPTLC methodology was successfully isolated (+)-catechin, (-)-epicatechin, (-)-epicatechin- gallate, procyanidin B1, procyanidin B2, procyanidin B3, proanthocyanidin B dimer gallate, emodin, emodin-8-*O*-glucoside, and emodin-8-*O*-malonyl-glucoside [239]. Fractionation or isolation using HPTLC has advantages such as a short separation time, pre- and post-chromatographic in-situ analyte derivatization, and crude extract can be applied without sample preparation [238,239]. The disadvantages of HPTLC are that it is relatively more expensive than TLC, and separation occurs only up to a certain length due to the limited plate length.

**Figure 5:**
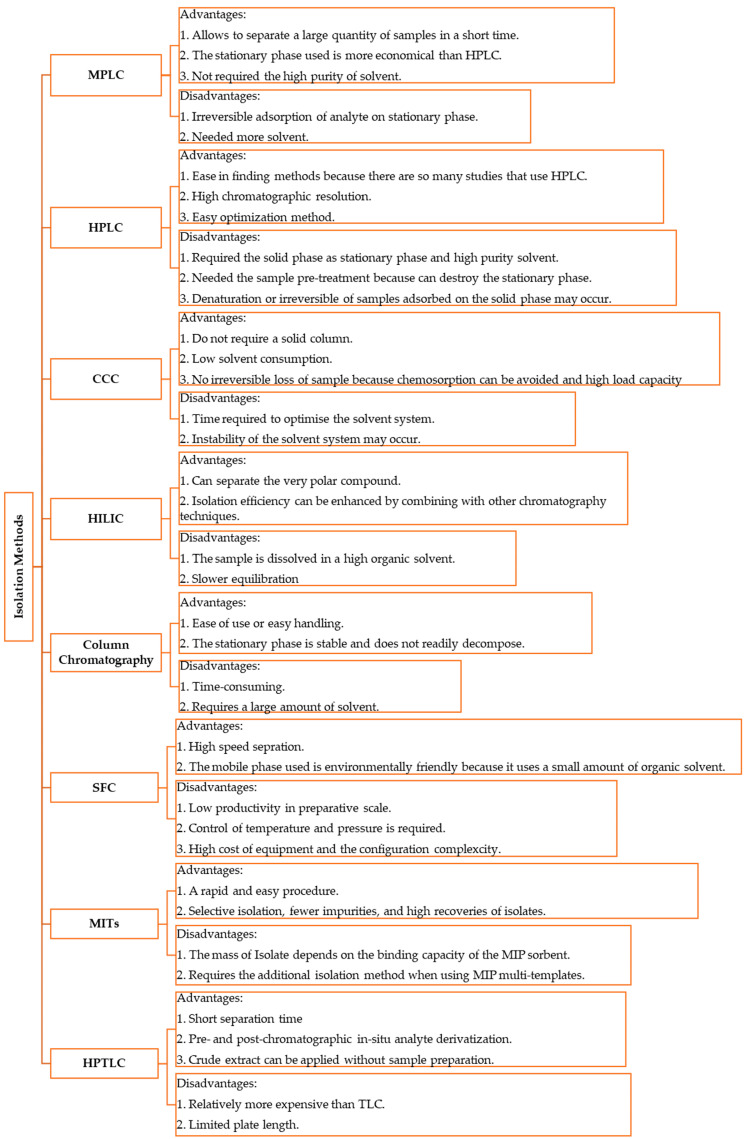
The advantages and disadvantages of isolation methods with new improvement from 2017 to 2023 to isolate antioxidant phenolic compounds.

To summarize the discussion on various separation methods used in the isolation of active compounds in natural products, Figure 5 provides the advantages and disadvantages of each method. 

## 4. Conclusions

Developing isolation methods for phenolic compounds with antioxidant activity is still exciting. Several methods that have been developed, such as MPLC, HPLC, HILIC, 2D-LC, CCC, column chromatography, SFC, MITs and HPTLC, have shown promising results with high compound purity >90%. Based on the advantages and disadvantages of different methods, it has been found that HPTLC, CCC, and MITs use less solvent than other methods. In terms of time, HPTLC and MITs are faster than other methods. Despite this, chromatography columns are still commonly used for the separation of phenolic compounds as they are cost-effective, easy to use, and do not require additional instruments. HPLC and MPLC can be combined with online DPPH detection to isolate phenolic compounds that have antioxidant activity. This combination helps to simplify the identification and isolation process of target compounds. Moreover, the CCC instrument can be guided by online DPPH HPLC to help isolate the antioxidant compound. Separation and purification using MITs may be further developed because this method is straightforward and selective. MIPs can be used directly on sample extracts; therefore, the fractionation process can be eliminated. 

Further investigation still needs to be conducted for a more efficient method with high purity and yield. There are several areas of potential research foci: compare several isolation methods for bioactive compounds in the same sample to determine the best and most effective method and studies related to the cost of isolation from each method to decide which method has the lowest cost with the highest % yield and %purity. In addition, it is necessary to develop a one-step procedure to streamline the isolation processing time. Finally, to promote the sustainable growth of green chemistry, it is required to modify the extraction and isolation solvents using less toxic, less hazardous, and environmentally friendly materials. 

## Figures and Tables

**Table 1:** The separation and purification methods used to isolate bergenin.

Sample	Separation and Purification Method	Yield (%) *	Purity (%)	Ref.
*Saxifraga atrata*	Polyamide column coupled with MCI GEL^®^ CHP20P in MPLC	1.9	>99	[39]
*Flueggea virosa* leaves	vacuum liquid chromatography column	2	NM	[86]
*Securinega virosa*	Silica column chromatography and Sephadex LH gel filtration chromatography	0.043	NM	[87]
*Peltophorum pterocarpum*	Crystallization	1	NM	[88]

NM, not mentioned in the article, * Yield (%): ratio of isolate mass and crude extract mass.

**Table 2:** Application of MPLC to isolate active compounds from natural products.

Sample	Compound	Separation Method	Yield (%) *	Purity (%)	Ref.
*Saxifraga atrata*	Bergenin	MPLC	1.9	>99	[39]
*Saxifraga atrata*	Ethyl gallate	MPLC-HPLC	0.013	>95	[89]
	11-*O*-Galloylbergenin		0.031		
	Rutin		1.12		
	Isoquercitrin		0.176		
*Saxifraga sinomontan*	3-methoxy-4-hydroxyphenol-(60-*O*-galloyl)-1-*O*-β-D-glucopyranoside	MPLC-RP-HPLC	0.6	>95	[90]
	3,4,5-trimethoxyphenyl-(60-*O*-galloyl)-1-*O*-β-D-glucopyranoside		1.39		
	Saximonsin A		1.40		
	Saximonsin B		0.24		

MPLC, medium-pressure liquid chromatography; RP-HPLC, reverse-phase high-performance liquid chromatography, * Yield (%): ratio of isolate mass and crude extract mass.

**Table 4:** Studies that have used high-speed counter-current chromatography (HSCCC) and high-performance counter-current chromatography (HPCCC) to isolate or purify phenolic compounds.

Sample	Compound	Instrument	Elution System	Yield (%) *	Purity (%)	Ref.
*Entada phaseoloides*	Phaseoloidin	HSCCC	*n*-Butanol:acetic acid:water, 4:1:5 (*v*/*v*)	7.76%	99.3%	[136]
	Entadamide A			6.97%	96.4%	
	Entadamide A-β-D-glucopyranoside			6.79%	97.7%	
*Malus hupehensis*	Avicularin	HSCCC	DES: choline chloride/glucose:water:ethyl acetate, 1:1:2 (*v*/*v*)	NM	93.1%	[41]
	Phloridzin				94.5%	
	Sieboldin				93.6%	
Sweet orange peel extract	Sinensetin	HPCCC	Hexanes:ethyl acetate:methanol:water, 1.4:0.6:1.4:0.6 (*v*/*v*), for the normal phase Hexanes:ethyl acetate:methanol:water, 0.7:1.3:0.7:1.3 (*v*/*v*), for the reverse phase	1.08%	100%	[42]
	3,5,6,7,3′,4′-hexamethoxyflavone			1.17%	100%	
	Nobiletin			14.25%	99.1%	
	5,6,7,4′-tetramethoxyflavone			2.17%	96.6%	
	3,5,6,7,8,3′,4′-heptamethoxyflavone			10.08%	98.4%	
	Tangeretin			2.75%	97.6%	
	(+)-catechin-phloroglucinol derivative;				76%	
	(-)-epicatechin				93%	
	(+)-catechin				77%	
	(-)-epicatechin-3-O-gallate				85%	
	(-)-epicatechin-3-O-galloyl-phloroglucinol derivative				95%	
*Parastrephia lucida* (Meyen)	11- p-coumaroyloxyltremetone	HSCCC	N-hexane: ethyl acetate:methanol:water (6:5:6:3 *v*/*v*/*v*)	11%	NM	[137]
Mango flowers	Gallic acid	HSCCC	N-hexane-ethyl acetate-methanol-water (4:6:4:6, *v*/*v*) for normal phase	1.85%	98.87%	[138]
	Ethyl gallate			1.95%	99.55%	
	Ellagic acid		dichloromethane-methanol-water (4:3:2, *v*/*v*) elution-extrusion mode	2.85%	99.71%	
*Achyrocline satureioides* (Lam) D.C.	Quercetin	HPCCC semi-preparative HPLC	N-hexane:ethyl acetate:methanol:water (0.8:1.0:0.8:1.0) and dichloromethane:methanol:water (3.5:3.5:2.5)	60%	97.5%	[139]
	Luteolin				90.2%	
	3-O-methylquercetin			65%	97.0%	
Peanut Hull	Luteolin	HPCCC	N-hexane:ethyl acetate:methanol:water (1.0:1.0:1.0:1.5)	1.5%	96%	[140]
	Eriodictyol			0.8%		
	5,7-dihydroxychromone			0.3%	99%	
Roots of *Polygonum multiflorum* Thunb	Gallic acid	HSCCC and preparative HPLC	Petroleum ether:ethyl acetate:methanol:water (1:5:1:5) Preparative HPLC: using methanol/water	NM	98.28%	[141]
	Epicatechin				96.71%	
	Piceatannol				96.85%	
	Rutin				97.92%	
	Resveratrol				96.94%	
	Hyperoside				98.52%	
Roots of *Polygonum multiflorum* Thunb	Catechin	HSCCC	Petroleum ether:ethyl acetate:methanol:water (1:5:1:5)	NM	90.69%	[141]
	Polydatin				94.91%	
	2,3,5,4′ -tetrahydroxy stilbene-2-O-β-D-glucoside				95.23%	
Leaves of *Lonicera japonica* Thunb.	Rhoifolin	HSCCCC	Methyl tert-butyl ether:n-butanol:acetonitrile:water (0.5% acetic acid) (2:2:1:5, *v*/*v*)	2.15%	94.3%	[142]
	Luteoloside			3.19%	96.1%	
	Chlorogenic acid	HSCCC and preparative HPLC	HSCCC system:methyl tert-butyl ether:n-butanol:acetonitrile:water (0.5% acetic acid) (2:2:1:5, *v*/*v*) Preparative HPLC system: C-18 (15 μm) column as stationary phase and solution of eluent A (methanol) and eluent B (0.3%, *v*/*v*, acetic acid in water) as mobile phase	1.09%	99.5%	
	Lonicerin			3.07%	98.7%	
	Rutin			1.67%	99.3%	
	3,4-O-dicaffeoylquinic acid			2.03%	97.1%	
	Hyperoside			1.82%	97.4%	
	3,5-O-Dicaffeoylquinic acid			2.47%	96.9%	
	4,5-O-Dicaffeoylquinic acid			2.61%	97.8%	
Persimmon	Gallic acid	HSCCC	N-hexane:ethyl acetate:water (3:17:20, *v*/*v*/*v*) and ethyl acetate:methanol:water (50:1:50, *v*/*v*/*v*)	3.13%	>95%	[143]
	Methyl gallate			29.47%		
	Epigallocatechin-3-gallate-(4β → 8, 2β → O → 7)-epigallocatechin-3-gallate dimer			3.93%		
*Salvia Miltiorrhiza*	Rutin	HSCCC	tert-butyl methyl ether/n-butanol/acetonitrile/water (3:1:1:20, *v*/*v*)	0.14%	97.3%	[144]
	Isoquercitrin			0.17%	99.5%	
*Mahonia bealei* (Fort.) Carr. Leaves	Chlorogenic acid	HSCCC	n-hexane/ethyl acetate/methanol/water (1:5:1:5, *v*/*v*/*v*/*v*)	NM	>92%	[145]
	Quercetin-3-O-β-D-glucopyranoside					
	Isorhamnetin-3-O-β-D-glucopyranoside					
*Castanopsis chinensis* Hance	Chinensin D	Combined multi step CC and HSCCC	N-Hexane/Ethyl acetate/Methanol/Water (1:6:3:4, *v*/*v*/*v*/*v*)	NM	93%	[134]
	chinensin E				95.7%	
*Chrysanthemum morifolium* cv. Fubaiju	Luteolin-7-O-β-D-glucoside	HSCCC combined with preparative HPLC	Ethyl acetate-n-butanol–acetonitrile–water–acetic acid (5:0.5:2.5:5:0.25, *v*/*v*/*v*/*v*/*v*)	NM	97.1%	[146]
	Luteolin-7-O-β-Dglucuronide				97.8%	
	Apigenin-7-O-β-D-glucoside				95.8%	
	luteolin- 7-O-β-D-rutinoside				96.7%	
	3,5-dicaffeoylquinic acid				97.8%	
	4,5-dicaffeoylquinic acid				97.5%	

NM, not mentioned in the article. * Yield (%): ratio of isolate mass and crude extract mass.

**Table 5:** A list of studies that used the HILIC.

Sample	Compound	Mode Separation	Purity	Yield(%) *	Ref.
*Saxifraga tangutica*	Hyperoside	2D HILIC/RPLC	>95%	NM	[154]
	Luteoline-glucoside				
	Trifolin				
*Salvia prattii*	Caffeic Acid	2D HILIC/RPLC	>98%	1.39	[155]
	Ethyl Rosmarinate			1.49	
	Methyl Rosmarinate			1.09	
	Rosmarinic acid			8.90	
*Dracocephalum heterophyllum*	caffeoyl-β-D glucopyranoside	2D RP/HILIC technique guided by on-line HPLC-DPPH	>95%	0.011	[153]
	ferruginoside B			0.014	
	verbascoside			0.083	
	2′-O-acetylplantamajoside			0.039	
	sibiricin A			0.026	
	luteolin			0.0106	
	rosmarinic acid			0.108	
	methyl rosmarinate			0.017	
*Arenaria* *kansuensis*	Tricin	2D RP/HILIC technique guided by on-line HPLC-DPPH	>98%	NM	[156]
	Homoeriodictyol				
	Luteolin				
*Lycium ruthenicum* Murr.	Anthocyanin	2D RP/HILIC	NM	NM	[156]

NM, not mentioned in the article. * Yield (%): ratio of isolate mass and crude extract mass.

## Data Availability

Data sharing is not applicable, no new data were created in this study.

## Supplementary Materials

The following supporting information can be downloaded at: https://www.mdpi.com/article/10.3390/plants13070965/s1, Figure S1: The mechanism linking inflammation to cancer; Figure S2: The Schematic of MPLC; Figure S3: Two schemes of isolation process of rutin. DPPH, 1,1-diphenyl-2-picrylhydrazyl; MPLC, medium-pressure liquid chromatography; RP-HPLC, reverse-phase high-performance liquid chromatography; Figure S4: Instrumentation of closed loop recycle-HPLC.

## Abbreviations

2D-LC	2-dimensional liquid chromatography
2D RP/HILIC	2-dimensional reversed-phase/hydrophilic interaction liquid chromatography
ABTS	3-ethylbenzothiazoline-6-sulfonic acid
ACN	Acetonitrile
DES	Deep eutectic solvent
DPPH	1,1-diphenyl-2-picrylhydrazyl
EGDMA	Ethylene glycol dimethacrylate
FRAP	Ferric reducing antioxidant power
TAC	Total antioxidant capacity
HPLC	high-performance liquid chromatography
MPLC	Medium-pressure liquid chromatography
CCC	Counter-current chromatography
CPC	Centrifugal partition chromatography
CUPRAC	Cupric reducing antioxidant capacity
HILIC	Hydrophilic interaction chromatography
HSCCC	High-speed counter-current chromatography
HPCCC	High-performance counter-current chromatography
HPTLC	High-performance thin layer chromatography
IC_50_	Half-maximal inhibitory concentration
MITs	Molecularly imprinted polymer techniques
MIP	Molecularly imprinted polymer
MIDSPE	Molecularly imprinted-dispersive solid-phase extraction
MISPE	Molecularly imprinted-solid phase extraction
MMIP	Magnetic Molecularly imprinted polymer
MMA	Methacrylic acid
Prep-HPLC	Preparative high-performance/high-pressure liquid chromatography
RNS	Reactive nitrogen species
ROS	Reactive oxygen species
RP-HPLC	Reverse-phase high-performance liquid chromatography
SFC	Supercritical fluid chromatography
SMIT	Surface molecularly imprinted technique
