# Supplementary material for: Separation Methods of Phenolic Compounds from Plant Extract as Antioxidant Agents Candidate
Source: Plants (Basel). 2024 Mar 27;13(7):965. doi: 10.3390/plants13070965 (PMC11013868; doi:10.3390/plants13070965)
Supplement: Supplementary file 1 [file plants-13-00965-s001.zip › plants-2914087-supplementary.pdf]

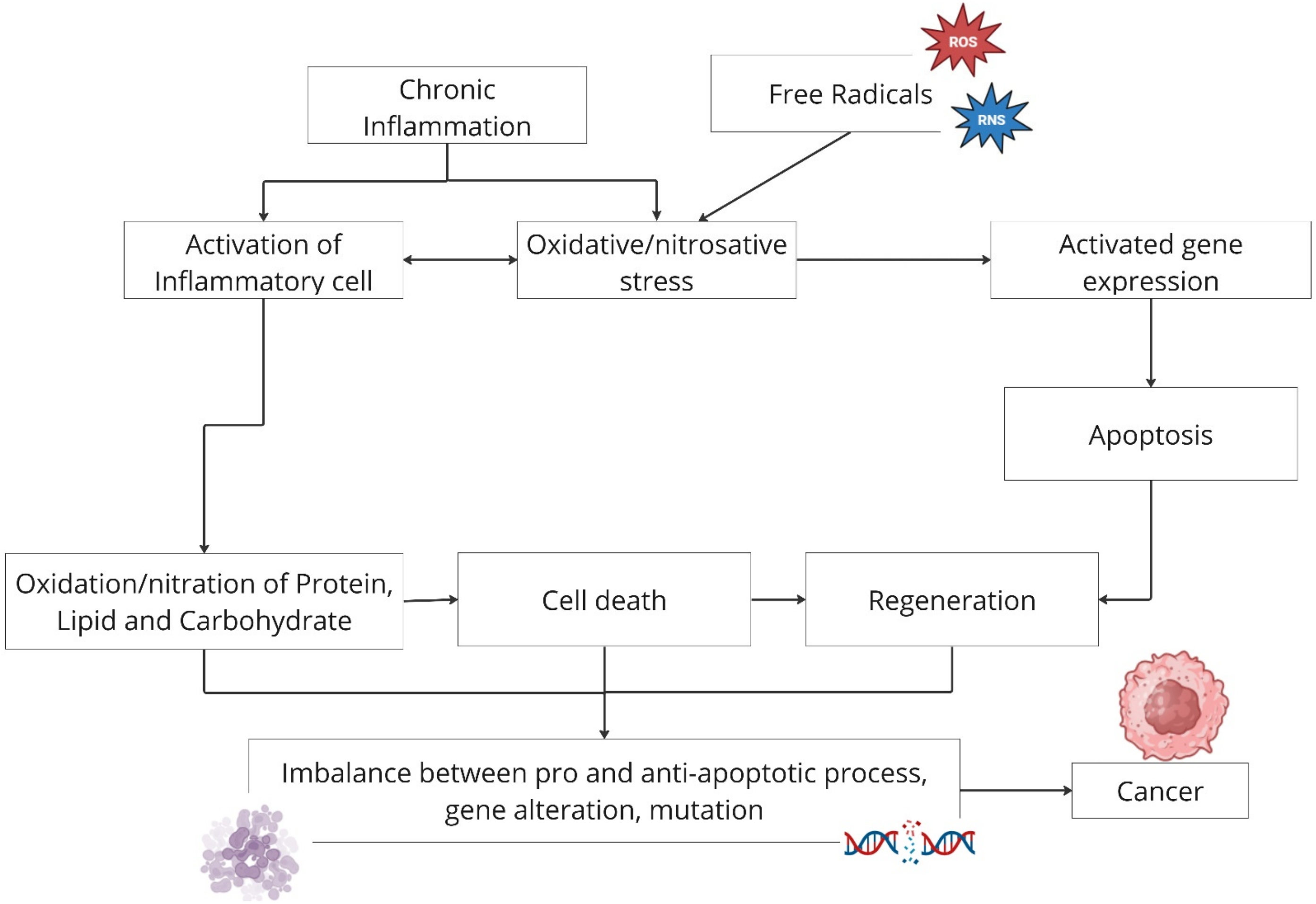

Figure S1. The mechanism linking inflammation to cancer.

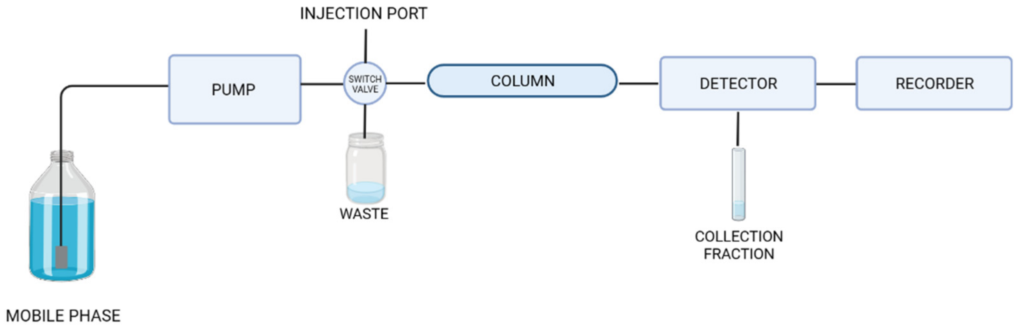

Figure S2. The Schematic of MPLC.

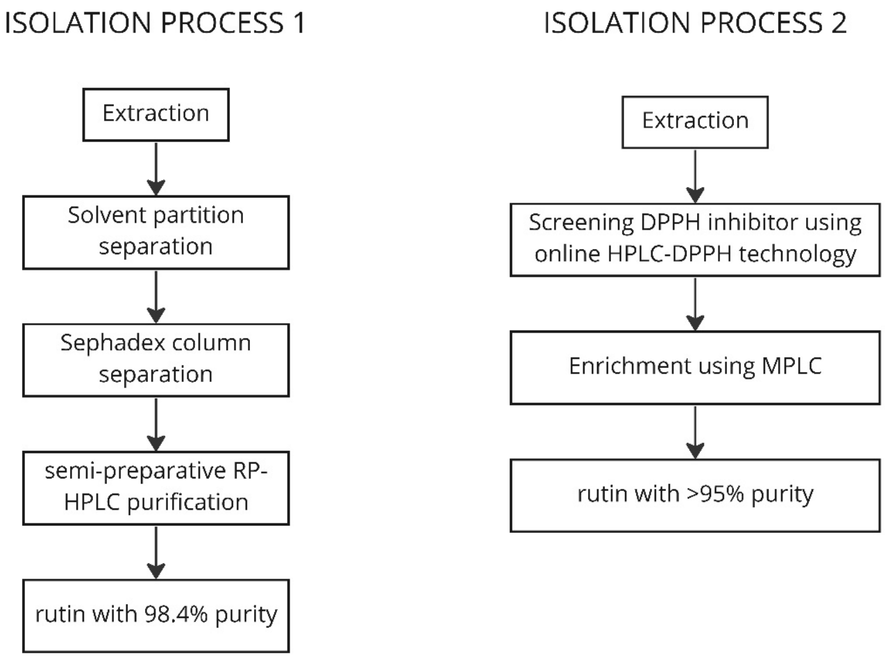

Figure S3. Two schemes of isolation process of rutin. DPPH, 1,1-diphenyl-2-picrylhydrazyl; MPLC, medium-pressure liquid chromatography; RP-HPLC, reverse-phase high-performance liquid chromatography.

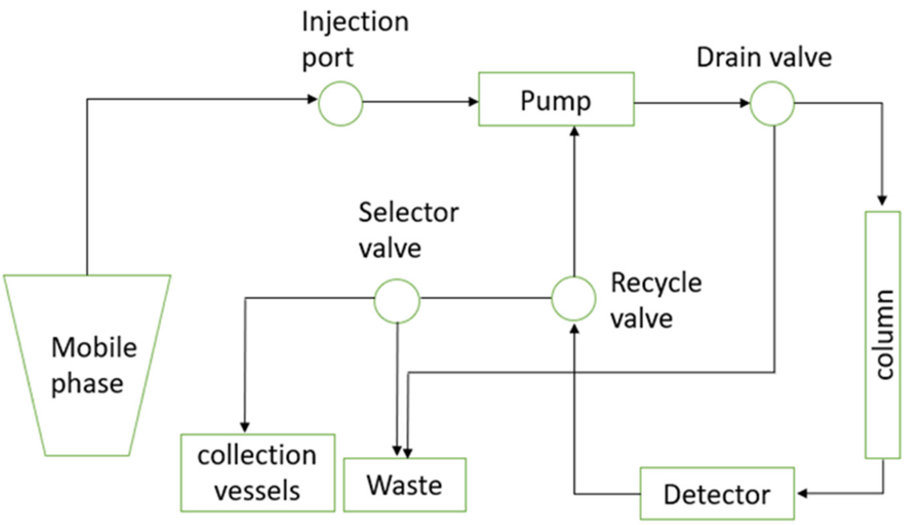

Figure S4. Instrumentation of closed loop recycle-HPLC.
